# Decreased Physical Activity and Endurance Capacity in Patients Qualified for Haematopoietic Stem Cell Transplantation (HSCT)

**DOI:** 10.3390/jcm14010186

**Published:** 2024-12-31

**Authors:** Michał Chmielewski, Agnieszka Szeremet, Małgorzata Stefańska, Paula Jabłonowska-Babij, Maciej Majcherek, Anna Czyż, Tomasz Wróbel, Iwona Malicka

**Affiliations:** 1 Active Recovery, Non-Profit Foundation, 53-019 Wroclaw, Poland; 2 Department and Clinic of Hematology, Cellular Therapies and Internal Medicine, Wroclaw Medical University, 50-367 Wroclaw, Poland; agnieszka.szeremet@umw.edu.pl (A.S.); paula.jablonowska@usk.wroc.pl (P.J.-B.); maciej.majcherek@umw.edu.pl (M.M.); a.czyz@umw.edu.pl (A.C.); tomasz.wrobel@umw.edu.pl (T.W.); 3 Department of Physiotherapy, Wroclaw University of Health and Sport Sciences, 51-612 Wroclaw, Poland; malgorzata.stefanska@awf.wroc.pl (M.S.); iwona.malicka@awf.wroc.pl (I.M.)

**Keywords:** physical activity, endurance capacity, haematopoietic stem cell transplantation, prehabilitation, haematology

## Abstract

**Background:** Haematological malignancies and their treatment regimens often lead to various complications that impair patients’ physical functioning. This study aimed to assess the level of physical activity and exercise capacity in patients with haematological malignancies who were qualified for haematopoietic stem cell transplantation (HSCT). **Methods:** A prospective, single-centre study was conducted on patients with haematological malignancies qualified for HSCT (study group, *n* = 103) and a cohort of healthy volunteers (reference group, *n* = 100). The assessment protocol included the International Physical Activity Questionnaire (IPAQ) and the 6-Minute Walk Test (6MWT). **Results:** The median age was 57 years in the study group and 56 years in the reference group. In the IPAQ assessment, at least 50% of the study group reported no engagement in moderate or intense physical activity. In the 6MWT, the study group demonstrated a significantly shorter walking distance compared to the reference group (*p *< 0.0001). Factors such as group membership (study vs. reference group), age, gender, and body mass index (BMI) were found to have a significant impact on 6MWT performance. No significant differences were observed in IPAQ or 6MWT results among subgroups within the study group when categorized by diagnosis. **Conclusions:** Patients with haematological malignancies who qualified for HSCT often show physical activity levels below recommended standards, which can negatively impact their ability to endure physical exertion. Insufficient activity prior to transplantation may contribute to reduced exercise capacity. Therefore, prehabilitation programmes aimed at improving physical activity and structured exercise should be an integral part of their care.

## 1. Introduction

In patients with haematological malignancies, complex treatment regimens are often administered, which significantly affect both their physical health and overall functioning [1]. Therapeutic procedures commonly used in these conditions, including chemotherapy, radiotherapy, and haematopoietic stem cell transplantation (HSCT), are essential for treating the underlying disease but are also associated with numerous adverse events (AEs) [2,3].

These side effects frequently result in reduced physical activity and diminished physical capacity, both of which are critical factors influencing a patient’s general health and their ability to tolerate ongoing anticancer treatment [4]. The combination of multifaceted cancer-related factors—such as anaemia, weakened immune function, impaired tissue regeneration, increased catabolism, and metabolic abnormalities (which may lead to cachexia)—with the adverse effects of anticancer therapies can contribute to a decline in physical fitness, making it difficult for patients to engage in regular physical activity before, during, and after treatment [5,6,7].

Physical activity encompasses all forms of movement requiring energy expenditure, driven by skeletal muscle contractions. This includes not only structured exercises and sports but also daily activities such as walking, household chores, and commuting. The World Health Organization (WHO) and the American College of Sports Medicine (ACSM) recommend at least 150 min of moderate-intensity physical activity or 75 min of vigorous-intensity activity per week to achieve significant health benefits [8,9]. Physical activity has long been recognized as one of the essential components for maintaining overall health and fitness, particularly among individuals coping with chronic diseases and/or oncological disease [10,11]. Studies have shown that regular exercise, especially aerobic training, improves cardiovascular function, muscle strength, and overall physical fitness, all of which are crucial for preserving a patient’s physical capacity and quality of life [12,13,14]. In the context of patients undergoing HSCT, physical fitness levels prior to the procedure, specifically before the conditioning phase, are of particular importance. Baseline fitness levels can significantly influence not only a patient’s ability to tolerate the transplantation process itself but also the speed and quality of recovery post-procedure. Higher levels of physical fitness prior to transplantation may not only enhance the body’s resilience to the stresses of the transplant but also contribute to shorter recovery times and a reduction in post-transplant complications [15,16,17,18].

HSCT, also referred to as bone marrow transplantation (BMT), is a physically intensive procedure, with its success heavily reliant on the physical fitness of patients eligible for the treatment. Patients undergoing BMT undergo a preparatory phase known as conditioning, which may involve whole-body irradiation or bone marrow ablation using high-dose chemotherapy (HDC). Following this phase, haematopoietic stem cells are administered intravenously. These stem cells can be sourced either from a compatible donor (allogeneic HSCT) or from the patient’s own previously collected cells (autologous HSCT) [3]. Reduced physical fitness prior to the initiation of transplantation may contribute to a prolonged recovery period, an increased risk of complications, and worsened overall treatment outcomes. Therefore, assessing and optimizing patients’ physical activity levels before transplantation is of critical importance, as it can be a significant factor in supporting treatment efficacy [19,20]. Incorporating prehabilitation, including structured physical exercise programmes, may serve as an effective strategy to enhance patients’ functional capacity and bolster physiological resilience. This approach has the potential to improve patients’ ability to endure the physical demands of HSCT, reduce the incidence of complications, and ultimately contribute to better long-term therapeutic outcomes [21,22].

Despite the well-documented benefits of regular physical activity, comprehensive data on physical activity levels and functional capacity in patients with haematological malignancies preparing for HSCT remain limited. Understanding how this specific patient population compares to healthy individuals in terms of physical activity and fitness is essential for developing optimized interventions tailored to their unique needs [15,23,24]. It is particularly important to investigate whether patients with haematological malignancies engage in sufficient physical activity to maintain adequate physical fitness, as well as to understand how limitations in activity affect their capacity for physical exertion. Such data could contribute to a better understanding of the needs of this patient group and support the development of strategies to aid in their preparation for a transplantation.

The International Physical Activity Questionnaire (IPAQ) was developed to assess individuals’ physical activity levels across various domains of life, such as occupational tasks, household duties, and leisure activities [25]. The IPAQ requires no specialized equipment, making it a highly accessible tool for both clinical and research use. It enables the categorization of an individual’s physical activity into three intensity levels, high, moderate, and low, thus offering valuable data on overall physical activity levels. The IPAQ’s simplicity and standardized global use make it ideal for both large-scale population studies and routine clinical assessments. Information gathered from this tool serves as a foundation for lifestyle interventions and the development of targeted rehabilitation strategies. As a result, the IPAQ is widely used in public health research and clinical practice as a reliable measure of physical activity [25,26,27].

The 6-min Walk Test (6MWT) is an objective tool used to assess exercise tolerance, providing essential insights into a patient’s physical capacity. It is especially valuable for evaluating functional endurance in patients with chronic conditions, such as respiratory and cardiovascular diseases, as well as those undergoing cancer treatments. The test delivers valuable information on a patient’s ability to tolerate physical activity, making it an effective tool for tracking changes in health status over time. Due to its versatility and minimal equipment requirements, the 6MWT has gained wide application in clinical practice, serving both to assess health status and to aid in planning and monitoring rehabilitation [28,29,30,31]. Its predictive value allows healthcare professionals to strategically plan, guide, and control the effects of physical activity and exercise interventions [32,33].

This study was designed and conducted to evaluate the level of physical activity and exercise capacity in patients with haematological malignancies who were qualified and prepared to undergo either autologous or allogeneic HSCT. The primary aim was to compare the physical activity levels of these patients with those of healthy individuals and to investigate the relationship between physical activity and exercise capacity, as measured by both the IPAQ and the 6MWT. Additionally, the study aimed to assess the impact of demographic factors, including gender, age, and body mass index (BMI), on the observed differences in physical fitness. It was hypothesized that patients eligible for HSCT exhibit lower levels of physical activity and reduced exercise capacity compared to healthy individuals.

## 2. Materials and Methods

This prospective study involved 103 patients (55 women, 48 men) diagnosed with haematological malignancies, treated at the Department of Haematology, Cellular Therapies, and Internal Medicine at the Medical University of Wrocław, all of whom were qualified for HSCT and constituted the study group. The reference group consisted of 100 healthy individuals (52 women, 48 men) with no history of cancer or comorbidities potentially affecting physical fitness.

In the study group, the median age was 57 years, with a median height of 170 cm, median weight of 77 kg, and a median of BMI of 27.12 kg/m^2^. In the reference group, the median age was 56 years, with a median height of 170 cm, median weight of 75.5 kg, and a median BMI of 25.95 kg/m^2^ (Table 1).

The study group was stratified into 6 diagnostic subgroups based on the underlying haematological condition: acute leukaemias (ALs), including acute myeloid leukaemia (AML) and acute lymphoblastic leukaemia (ALL) (*n* = 29); myelodysplastic neoplasms (MDSs, *n* = 7); myeloproliferative neoplasms (MPNs, *n* = 11); lymphomas, encompassing non-Hodgkin lymphoma (NHL) and Hodgkin lymphoma (HL) (*n* = 21); plasma cell myelomas (PCMs, *n* = 32); and a category designated as “other”, which included severe aplastic anaemia (SAA, *n* = 3) [34].

This study was conducted based on clearly defined inclusion and exclusion criteria for both groups (detailed below).

For the study group, the inclusion criteria were as follows: adults aged up to 75 years, a confirmed diagnosis of a haematological malignancy, an established indication for HSCT, eligibility for the procedure, and submission of a written informed consent form (ICF) to participate in the study.

For the reference group, the inclusion criteria included adults aged up to 75 years, good overall health, no history of malignant neoplasms or chronic diseases, and no use of long-term medications.

The exclusion criteria for both groups included comorbidities significantly impacting physical activity and fitness, intellectual or motor disabilities, and lack of informed consent to participate in the study.

A total of 220 individuals were initially recruited for the reference group. To ensure a sample size comparable to the study group, 100 participants were randomly selected using an online random number generator (https://generatorliczb.pl/, accessed on 10 July 2024). The results from these selected individuals were subsequently included in the final analyses.

## 3. Research Methods

This study was conducted between September 2022 and December 2023. As part of the research protocol, participants were required to complete the IPAQ-SF and undergo the 6MWT. Both assessments were administered by qualified medical personnel following the participants’ admission to the transplant unit and prior to the initiation of treatment.

The Supplementary Materials IPAQ Short Form (IPAQ-SF) was utilized to assess subjects’ physical activity levels. This standardized tool comprises seven questions addressing various domains of physical activity, including work-related tasks, leisure activities, and daily routines. Participants completed the questionnaire after receiving detailed instructions and clarification to ensure the accuracy and reliability of their responses. Only activities performed within the preceding seven days, sustained for a minimum duration of 10 min without significant interruptions, were recorded. The IPAQ evaluates individual physical activity levels using Metabolic Equivalent of Task (MET) units, which quantify energy expenditure as a multiple of the resting metabolic rate. One MET corresponds to the energy expended while at rest. Physical activities are assigned standard MET values: walking is rated at 3.3 METs, moderate-intensity activities at 4.0 METs, and vigorous-intensity activities at 8.0 METs. The total MET score for each activity type is calculated by multiplying its assigned MET value by the frequency of activity per week (days) and the duration per day (minutes). Weekly MET scores are then summed across all activities to determine overall physical activity levels. Based on the cumulative MET-min/week score, individuals are categorized into one of three groups: high physical activity (>3000 MET-min/week), moderate physical activity (600–3000 MET-min/week), or low physical activity (<600 MET-min/week) [35].

The 6MWT was used as an indicator of exercise capacity in this study. The test was conducted in an enclosed space on a flat, 30-metre-long surface. Participants were instructed to walk the longest possible distance within six minutes, maintaining a steady walking pace without running or jogging, and turning at each end of the corridor to ensure continuity. Blood pressure and heart rate were measured before and after the test for all subjects. If necessary, participants were permitted to slow down or rest by sitting in a chair during the test, with the timer continuing to run. The total distance (in metres) covered during the test was recorded.

### 3.1. Ethics

All participants provided written ICF before enrolment in the study. The research was conducted in full compliance with the principles outlined in the Declaration of Helsinki and received ethical approval from the Wroclaw Medical University Ethics Committee (approval No.: KB-843/2021; approval date: 28 October 2021).

### 3.2. Statistical Analysis

The Shapiro–Wilk test was used to check the distribution of all analysed variables. Due to the non-normality of the distribution of most of the analysed characteristics, the median was used as a measure of central tendency and the interquartile range (IQR) as a measure of dispersion. The significance of differences between groups was tested using the Man–Whitney U test, χ^2^ or Kruskal–Wallis ANOVA with Dunn Bonferroni–Holm post hoc test. Multiple regression models were created to determine the significance of the effect of the selected variables on the level of exercise capacity tested by the 6MWT. In each regression analysis, the normality of the distribution of the residuals was confirmed. The effect size for the Man–Whitney U test and Kruskal–Wallis ANOVA were estimated using Eta-squared (η^2^) transformed to Cohen’s d coefficient value [36]. Calculations were performed in Statistica 13.1 (TIBCO Software Inc., Palo Alto, CA, USA), PQStat 1.8.2 (PQStat Software, Poznań, Poland) and using the online effect measure calculator at https://www.psychometrica.de/effect_size.html (accessed on 12 July 2014). A value of *p *< 0.05 was taken as the significance level for all tests used.

## 4. Results

A summary of the IPAQ results revealed that at least 50% of participants in the study group reported no engagement in intense or moderate physical activity. Additionally, the average total IPAQ score in the study group was more than two times lower compared to the reference group. Significant differences were also observed between the groups in the 6MWT results. The median walking distance in the reference group was nearly 200 m longer than in the study group (Table 2).

An analysis of activity level scores in the group of haematology patients according to clinical diagnosis showed no significant differences between subgroups in the partial and total scores of the IPAQ and the 6MWT scores. The highest overall MET scores were observed in subgroups 3 (MPN) and 4 (NHL and HL), while the highest 6MWT scores were observed in subgroup 4 (NHL and HL). The lack of significance of differences between subgroups may be due to the underrepresentation of some subgroups, e.g., diagnosis 2—seven patients; diagnosis 6—three patients (Table 3 and Table 4). There was also no significant variation in the results describing the physical activity of the study group according to the treatment lines used and the presence or absence of chemotherapy in the course of previous treatment (Table 4).

The 6MWT score was taken as an indicator of exercise capacity. Using a multivariate regression model for all subjects, the test result was shown to be significantly influenced by group membership (study group vs. reference group), gender, age, and the subjects’ BMI level. A significantly shorter recorded 6 min distance was observed in the study group by an average of about 140 m in men and about 50 m in women. It was also observed that an increase in the age of the subjects by 1 year decreases the test result by less than 2 metres, while an increase in BMI by 1 kg/m^2^ results in a shortening of the distance by about 4 m (Table 5).

A multivariate regression model identifying predictors of exercise capacity in a group of haematology patients showed a significant effect of gender, patient age, and BMI on the 6MWT results. It was observed that the women’s group scored approximately 40 m shorter than the men’s group; additionally, age at the time of HSCT, higher by 1 year, reduced the achieved walking score by an average of 2.5 m, and an increase in BMI by 1 kg/m^2^ resulted in a decrease in the distance by more than 5 m. In addition, a significantly shorter distance walked was observed in patients with diagnosis 1 (AL) compared to the reference, which consisted of patients with lymphomas (Table 6).

## 5. Discussion

This study aims to evaluate the level of physical activity and its relationship with exercise capacity in patients with haematological malignancies qualified for HSCT.

Anticancer therapies are known to produce predictable side effects, significantly increasing the risk of neuromuscular and cardiovascular complications [37]. In addition, the treatment of haematological malignancies often leads to a range of symptomatic side effects, including physical and psychological pain, fatigue, insomnia, infections, polyneuropathy, appetite loss, and gastrointestinal mucositis. These symptoms not only hinder daily functioning but can also complicate rehabilitation protocols, delay treatment schedules, and, in some cases, necessitate premature discontinuation of therapy [38]. Given these challenges, optimizing patients’ physical fitness and exercise capacity prior to initiating intensive treatment is essential. Effective prehabilitation strategies can help mitigate adverse symptoms, improve treatment tolerance, and potentially enhance overall clinical outcomes.

In this study, IPAQ results revealed that at least 50% of patients qualified for HSCT demonstrated only a low level of physical activity. Compared to a reference group of healthy individuals, haematological patients scored, on average, 50% lower on the overall IPAQ, reflecting significantly reduced daily physical activity among those awaiting transplantation. Similar observations were reported by Asmita Mishra et al., where the majority of patients preparing for transplantation engaged primarily in light (36%) and moderate (43%) physical activity, with only 21% reporting high levels of physical activity [39].

Importantly, physical activity is widely acknowledged as an effective adjunctive therapy, offering proven health benefits and demonstrating a high safety profile [40]. Both the present study and the findings of Asmita Mishra et al. highlight that patients awaiting HSCT generally do not meet the physical activity guidelines set by the WHO and the ACSM [8,9].

In haematological malignancies, data regarding physical activity levels in patients prior to HSCT remains limited. On a broader scale, regardless of the specific type of cancer, patients generally exhibit lower levels of physical activity than recommended, with a further decline observed during treatment. A study by Büntzel et al. reported a decrease in physical activity from 71% before cancer diagnosis to 50% during treatment and 40% after treatment [41]. Similarly, many patients awaiting HSCT report low levels of physical activity, often attributing this to the intense pre-transplant regimen and disease-related symptoms [42]. The decline in physical activity can be linked to various factors, with the side effects of treatment being the most prominent contributors. The most commonly reported symptoms include fatigue (94.3%) and pain (75.6%) [38]. In a study by Craike et al., the primary barriers to physical activity in multiple myeloma patients were identified as fatigue (37.8%), pain (28.1%), and a lack of knowledge about safe physical exercises (19.7%). Interestingly, patients who maintained higher levels of physical activity prior to diagnosis were 4.79 times more likely to preserve adequate activity levels after treatment [43]. This observation highlights the significant role of pre-diagnosis physical activity as a strong predictor of post-treatment physical activity levels [44].

A lack of motivation can serve as a significant barrier to physical activity. Individuals who are naturally more physically active often demonstrate greater engagement in rehabilitation exercises, while reduced motivation is frequently associated with chronic fatigue and neuropathy [41]. In patients with haematological malignancies, physical weakness is a common concern, often arising from treatment-related side effects such as cachexia, neurotoxicity, and cardiotoxicity. Additionally, cancer-related fatigue (CRF) is exacerbated by comorbid conditions, including anaemia, neutropenia, and sarcopenia [32]. CRF often creates a vicious cycle, where physical weakness limits activity, further intensifying fatigue, which in turn contributes to greater physical limitations and an increased risk of mood disorders [45].

Granger emphasizes that the cytotoxic effects of chemotherapy have a profound impact on both exercise capacity and muscle strength, often surpassing the detrimental effects of reduced physical activity alone during treatment. Approximately 25% of patients with haematological malignancies experience cancer-related cachexia, while 51% exhibit sarcopenia even before treatment initiation. Both conditions lead to reduced muscle mass and strength, unintentional weight loss, and heightened fatigue, ultimately resulting in impaired physical fitness and a decline in overall functional capacity [44].

Newcomb et al. underscore the importance of the psychological dimension in the context of physical activity among patients. Compared to younger individuals, older patients may be more prone to exhibiting depressive symptoms, such as difficulty concentrating and chronic fatigue, often stemming from their illness and diagnosis. These symptoms can significantly affect daily functioning and reduce the capacity to engage in physical activity [46]. Similarly, Nakamura et al. highlight the influence of psychological factors, including anxiety, depression, and stress associated with both the diagnosis and treatment process. These factors can markedly diminish motivation and decrease the willingness to participate in physical activity, creating additional barriers to effective rehabilitation and recovery [47].

In this study, statistically significant differences were observed in the time spent sitting between the two groups. The median sitting time in the study group was 1500 min per week, compared to 300 min per week in the reference group. Similarly, in a study by Asmita Mishra et al., patients eligible for transplantation reported a median sitting time of 6 h per day, equating to approximately 2520 min per week [39]. Sweegers et al. also found that cancer survivors tend to spend the majority of their day sitting, with only minimal time dedicated to moderate to vigorous physical activity. Notably, survivors of haematological malignancies were significantly less likely to exhibit a highly active lifestyle compared to breast cancer survivors [48]. While the authors did not identify a specific cause for this discrepancy, they highlighted a clear association between fatigue levels and physical activity—patients with lower fatigue levels were more likely to maintain higher levels of physical activity. Fatigue is one of the most prevalent side effects reported by cancer patients, often emerging as a consequence of active anticancer treatment. It is almost universally experienced by individuals undergoing cytotoxic chemotherapy and bone marrow transplantation and is also commonly observed in patients receiving radiotherapy.

In this study, the 6MWT revealed statistically significant differences between the two groups, with participants in the study group covering approximately 200 m less than those in the reference group. The median distance walked was 412.50 m in the study group and 603.50 m in the reference group. Comparable findings have been reported in previous studies. Asmita Mishra et al. observed a median walking distance of 431 m among transplant-eligible patients [39]. Similarly, Jones et al. reported a median distance of 404 m in their study group, which represented 59% of the predicted distance based on age and gender [49]. In another study, Takekiyo et al. evaluated patients two weeks before transplantation and noted a median walking distance of 482 m in the 6MWT [50]. These findings collectively highlight the consistent reduction in exercise capacity among patients preparing for HSCT.

In contrast, Morishita et al. reported a median of the 6MWT distance of 520.4 m in patients with haematological malignancies, a value notably higher than that observed in the present study. This discrepancy may stem from differences in patient demographics, as the Morishita et al. study included younger, male-only participants, while both age and gender were found to significantly influence 6MWT outcomes in our study [51].

Furthermore, BMI emerged as a key predictor of exercise capacity, with higher BMI values associated with poorer functional outcomes. Notably, elderly patients with a BMI ≥ 30 kg/m^2^ are approximately 60% more likely to experience functional impairment compared to those with normal weight [52].

A multivariate regression model analysing predictors of exercise capacity within our cohort revealed that patients diagnosed with AL walked significantly shorter distances in the 6MWT compared to those diagnosed with lymphomas. This disparity may be attributed to the intensive chemotherapy regimens typically administered in AL treatment protocols. These regimens often involve high-dose cytotoxic agents, such as anthracyclines and cytarabine, aimed at achieving disease remission [53]. However, these high-intensity protocols frequently result in bone marrow suppression and prolonged aplasia, necessitating extended hospitalizations and a controlled environment to mitigate risks associated with infections and other complications [54,55]. Consequently, patients’ physical activity levels are markedly limited during this period. Moreover, the side effects of such treatments—including severe fatigue, muscle wasting, and cardiotoxicity—are well documented contributors to declines in physical functioning and reduced exercise tolerance [56]. In contrast, patients with lymphomas, particularly older individuals or those with comorbidities, may often undergo less intensive chemotherapy protocols or targeted therapies. These treatment approaches generally exhibit lower systemic toxicity, which can help better preserve physical endurance and muscle function [57].

The reduced exercise capacity observed in pre-transplant patients can be attributed to several factors, including muscle oxygen saturation levels and haemoglobin concentration. Morishita et al. demonstrated that patients awaiting transplantation often exhibit lower muscle oxygen saturation, which significantly impacts their 6MWT performance. The authors concluded that reduced muscle oxygen saturation may impair oxygen consumption and blood flow during physical exertion, thereby compromising exercise capacity [51]. Additionally, the prolonged treatment process, often involving glucocorticoid therapy with agents such as dexamethasone, further contributes to reduced physical performance. Dexamethasone induces glucocorticoid-mediated apoptosis, and its extended use (beyond 7 days) is associated with sarcopenia and insulin resistance, both of which lead to diminished muscle strength and functional capacity [58]. These limitations in physical activity and exercise capability are also strongly linked to the toxic effects of treatment, including the cytotoxic impact of chemotherapy [44,59]. Notably, even months before treatment initiation, patients with haematological malignancies may experience reduced muscle strength and decreased exercise capacity. These deficits are often linked to disease-related protein catabolism and elevated levels of tumour necrosis factor (TNF), which can contribute to muscle cachexia and abnormal muscle contractions [44]. Collectively, these findings emphasize the multifactorial nature of exercise capacity limitations in pre-transplant patients, highlighting the roles of muscle oxygenation, treatment-related toxicity, and disease-specific metabolic disturbances in shaping physical performance outcomes.

Any type of cancer and its associated treatment protocols lead to a decline in physical performance across all stages of the disease. This deterioration contributes to reduced functional independence and increased mortality rates [60]. HSCT is particularly intensive and physically demanding, requiring patients to maintain a robust functional status to endure the procedure and optimize outcomes [61]. Currently, patient eligibility for transplantation is primarily determined based on functional status and the presence of comorbidities, as evaluated by the attending physician. However, this assessment approach offers only a partial perspective on the patient’s overall health and resilience [62]. With the increasing age of transplant recipients [44], the pre-treatment functional status of patients has become an even more critical consideration. According to Jayani and Olin, 10–33% of older adults exhibit signs of frailty prior to transplantation. Additionally, 36% of all pre-transplant patients experience weakness syndrome, including 26% of younger adults. Patients presenting with weakness syndrome face a higher risk of post-transplant toxicity and lower overall survival (OS) rates [63]. Evidence suggests that physical functional status is a strong predictor of mortality and is closely correlated with symptom severity in patients with lymphoma, myeloma, and leukaemia [58].

### Limitations of the Study

The primary limitation of this study lies in the diversity of diagnoses among participants. To determine whether correlations differ based on specific types of haematological malignancies, larger subgroup samples would be required. Another limitation may arise from the IPAQ itself. While the short version used in this study is more user-friendly than the extended form, participants often encounter difficulties estimating the time spent on various activities. Such inaccuracies, including underestimation or overestimation of physical activity levels, can influence the final results of the questionnaire and, consequently, the study outcomes. Notably, the IPAQ-SF was previously validated in cancer patients undergoing treatment in a study by Vassbakk-Brovold et al. (2016). Their findings revealed that patients reported 366% higher levels of moderate to vigorous physical activity on the IPAQ compared to objective measurements [64]. Additionally, the absence of baseline assessments of cardiorespiratory fitness and blood morphotic parameters represents another limitation, as such data would provide a clearer basis for equivalence between the study and reference groups. Future research should emphasize a comprehensive medical evaluation of both groups to address these limitations effectively.

## 6. Conclusions

Patients eligible for HSCT typically exhibit lower levels of physical activity than those recommended by the WHO. Physical activity levels are closely linked to exercise capacity in this patient population, making prehabilitation programmes focused on structured exercise and increased physical activity a crucial element of comprehensive care for patients with haematological malignancies. The intensive nature of anticancer treatment often results in significant adverse effects, including sarcopenia, cachexia, and fatigue, all of which contribute to a decline in functional status. When compounded by low physical activity levels prior to transplantation, these factors lead to a statistically significant reduction in exercise capacity, posing a substantial challenge for this vulnerable group. To address these issues, pre-transplant education on physical activity should be considered an essential component of patient preparation. Furthermore, referring patients to qualified physiotherapists can enable the creation of personalized exercise programmes tailored to the individual’s capabilities and clinical needs. Further research is necessary to deepen our understanding of the factors contributing to reduced exercise capacity and to optimize prehabilitation and haematological rehabilitation protocols, ultimately improving outcomes for patients undergoing HSCT.

## Figures and Tables

**Table 1:** Characteristics of the groups.

		Study Group *n* = 103		Reference Group *n* = 100		U MW Test
		Median	IQR	Median	IQR	*p*
Age [years]		57.00	18.00	56.00	17.00	0.6181
Body height [cm]		170.00	14.00	170.00	12.50	0.4065
Body weight [kg]		77.00	26.00	75.50	16.50	0.6190
BMI [kg/m^2^]		27.12	6.82	25.95	5.57	0.2795
		n	%	n	%	χ^2^ p
Gender	Female Male	55	53	52	52	0.5636
		48	47	48	48	

Abbreviations: IQR—interquartile range; U MW test—Mann–Whitney U test; χ^2^—chi-squared test.

**Table 2:** Comparison of physical activity in the study and reference groups.

	Study Group *n* = 103		Reference Group *n* = 100		Test U MW	Cohen’s d
	Median	IQR	Median	IQR	*p*	
Intensive activities (min/week)	0.00	0.00	85.00	195.00	<0.0001 *	0.72
Moderate activities (min/week)	0.00	180.00	142.50	255.00	<0.0001 *	0.83
Walking (min/week)	150.00	525.00	240.00	345.00	0.2535	0.16
Sitting (min/week)	1500.00	1500.00	300.00	240.00	<0.0001 *	1.45
Intensive [MET]	0.00	0.00	680.00	1560.00	<0.0001 *	0.72
Moderate [MET]	0.00	720.00	570.00	1020.00	<0.0001 *	0.83
Walking [MET]	495.00	1732.50	792.00	1138.50	0.2535	0.16
IPAQ MET in general	1580.90	2772.50	2627.00	3370.00	0.0021 *	0.44
6 min walk test [m]	412.50	117.00	603.50	126.00	<0.0001 *	1.57

Abbreviations: IQR—interquartile range, * *p *< 0.05.

**Table 3:** Physical activity in the study group according to diagnosis.

	Diagnosis 1 *n* = 29		Diagnosis 2 *n* = 7		Diagnosis 3 *n* = 11		Diagnosis 4 *n* = 21		Diagnosis 5 *n* = 32		Diagnosis 6 *n* = 3	
	Median	IQR	Median	IQR	Median	IQR	Median	IQR	Median	IQR	Median	IQR
Intensive activities (min/week)	0	0	0	0	0	30	0	300	0	8	40	2520
Moderate activities (min/week)	0	120	0	0	120	120	60	540	0	128	30	1200
Walking (min/week)	210	555	150	690	120	1080	120	480	210	293	300	2055
Sitting (min/week)	1200	1800	1800	900	1500	1200	1500	1800	1223	900	2400	2400
Intensive [MET]	0	0	0	0	0	240	0	2400	0	60	320	20,160
Moderate [MET]	0	480	0	0	480	480	240	2160	0	510	120	4800
Walking [MET]	693	1832	495	2277	396	3564	396	1584	693	965	990	6782
IPAQ MET in general	1386	2277	1067	2277	2320	3282	2156	5844	1243	2337	1430	31,742
6 min walk test [m]	401	127	379	50	396	36	462	74	448	115	413	75

Diagnoses [1–6]: [1] Acute leukaemias (ALs); [2] myelodysplastic neoplasms (MDSs); [3] myeloproliferative neoplasms (MPNs); [4] lymphomas (NHL and HL); [5] plasma cell myeloma (PCM); [6] other; IQR—interquartile range.

**Table 4:** Significance of the effect of diagnosis, number of lines of treatment and chemotherapy on physical activity of the study group.

	Diagnoses 1–6		Treatment Lines: 1–3		Chemotherapy	
	ANOVA Kruskal–Wallis	Cohen’s	ANOVA Kruskal–Wallis	Cohen’s	Test U MW	Cohen’s
	*p*	d	*p*	d	*p*	d
Intensive activities (min/week)	0.0655	0.48	0.4050	0.09	0.9673	0.01
Moderate activities (min/week)	0.1966	0.31	0.2547	0.17	0.8714	0.03
Walking (min/week)	0.9504	0.41	0.2396	0.19	0.2459	0.23
Sitting (min/week)	0.2950	0.22	0.5343	0.17	0.0933	0.34
Intensive [MET]	0.0655	0.48	0.4050	0.09	0.9673	0.01
Moderate [MET]	0.1966	0.31	0.2547	0.17	0.8714	0.03
Walking [MET]	0.9504	0.41	0.2396	0.19	0.2459	0.23
IPAQ MET in general	0.8496	0.36	0.0714	0.37	0.4496	0.15
6 min walk test [m]	0.0666	0.48	0.5226	0.17	0.3806	0.20

Diagnoses [1–6]: [1] Acute leukaemia (AL); [2] myelodysplastic neoplasms (MDSs); [3] myeloproliferative neoplasms (MPNs); [4] lymphomas (NHL and HL); [5] plasma cell myeloma (PCM); [6] other. Lines of treatment [1–3]: [1]—0 or 1 line; [2]—2 lines; [3]—3 and more lines. Chemotherapy: [0]—no; [1]—yes.

**Table 5:** Predictors of the 6 min test score—all subjects.

Model: Dependent Variable—6 min Test Score					
	b	Error b	−95% CI	+95% CI	*p*-Value
Group: study [1], reference [0]	−139.03	20.32	−179.21	−98.86	<0.0001 *
Gender: male [0]; female [1]	−52.41	18.22	−88.43	−16.39	0.0047 *
Age [years]	−1.85	0.58	−2.99	−0.72	0.0016 *
BMI [kg/m]^2^	−4.08	1.74	−7.52	−0.64	0.0203 *
Walking (min/week)	0.00	0.01	−0.02	0.02	0.7849
Sitting (min/week)	−0.01	0.01	−0.02	0.01	0.5390 *
Activity level [1–3]	−0.07	11.35	−22.52	22.37	0.9949
R^2^ adjusted	0.37				
Standard error of estimate	88.19				
F	13.47				
The *p*-value	<0.0001 *				

Activity level [1–3]: [1]—low (<600 MET); [2]—moderate (600–3000 MET); [3]—high (>3000 MET); * *p *< 0.05.

**Table 6:** Predictors of the 6 min test score—study group.

Model: Dependent Variable 6 min Test Score						
	**b**	Error b	−95% CI	+95% CI	Stat. t	*p*-Value
Gender: male [0]; female [1]	−40.53	16.34	−73.00	−8.07	−2.48	0.0150 *
Time from diagnosis to transplant [months]	−0.34	0.36	−1.05	0.37	−0.96	0.3398
Age at transplantation [years]	−2.52	0.69	−3.90	−1.14	−3.63	0.0005 *
BMI [kg/m]^2^	−5.23	1.80	−8.80	−1.65	−2.90	0.0047 *
Diagnosis_group [1]	−48.18	23.38	−94.64	−1.72	−2.06	0.0423 *
Diagnosis_group [2]	−67.96	39.57	−146.58	10.65	−1.72	0.0893
Diagnosis_group [3]	−27.66	34.98	−97.17	41.84	−0.79	0.4312
Diagnosis_group [5]	2.99	22.16	−41.04	47.03	0.13	0.8929
Diagnosis_group [6]	−92.53	55.59	−202.98	17.92	−1.66	0.0995
Number of treatment lines	−6.74	12.50	−31.58	18.10	−0.54	0.5913
Chemotherapy: No [0]/Yes [1]	−1.09	33.63	−67.91	65.72	−0.03	0.9741
R^2^ adjusted	0.25					
Standard error of estimate	76.76					
F	4.09					
The *p*-value	0.0001 *					

Recognition: Reference category of 4 diagnoses [1–6]: [1] Acute leukaemias (ALs); [2] myelodysplastic neoplasms (MDSs); [3] myeloproliferative neoplasms (MPNs); [4] lymphomas (NHL and HL); [5] plasma cell myeloma (PCM); [6] other. Lines of treatment [1–3]: [1]—0 or 1 line; [2]—2 lines; [3]—3 or more lines. Chemotherapy: [0]—no, [1]—yes; * *p *< 0.05.

## Data Availability

The raw data supporting the conclusions of this article are available in an open access repository: https://zenodo.org/records/14541613.

## Supplementary Materials

The following supporting information can be downloaded at: https://www.mdpi.com/article/10.3390/jcm14010186/s1. The International Physical Activity Questionnaire-Short Form (IPAQ-SF).
